# *PACSIN2* polymorphism is associated with thiopurine-induced hematological toxicity in children with acute lymphoblastic leukaemia undergoing maintenance therapy

**DOI:** 10.1038/srep30244

**Published:** 2016-07-25

**Authors:** Alenka Smid, Natasa Karas-Kuzelicki, Janez Jazbec, Irena Mlinaric-Rascan

**Affiliations:** 1 Faculty of Pharmacy, University of Ljubljana, Ljubljana, Slovenia; 2 University Children’s Hospital, University Medical Centre Ljubljana, Ljubljana, Slovenia

## Abstract

Adequate maintenance therapy for childhood acute lymphoblastic leukemia (ALL), with 6-mercaptopurine as an essential component, is necessary for retaining durable remission. Interruptions or discontinuations of the therapy due to drug-related toxicities, which can be life threatening, may result in an increased risk of relapse. In this retrospective study including 305 paediatric ALL patients undergoing maintenance therapy, we systematically investigated the individual and combined effects of genetic variants of folate pathway enzymes, as well as of polymorphisms in *PACSIN2* and *ITPA*, on drug-induced toxicities by applying a multi-analytical approach including logistic regression (LR), classification and regression tree (CART) and generalized multifactor dimensionality reduction (GMDR). In addition to the TPMT genotype, confirmed to be a major determinant of drug related toxicities, we identified the *PACSIN2* rs2413739TT genotype as being a significant risk factor for 6-MP-induced toxicity in wild-type TPMT patients. A gene-gene interaction between *MTRR* (rs1801394) and *MTHFR* (rs1801133) was detected by GMDR and proved to have an independent effect on the risk of stomatitis, as shown by LR analysis. To our knowledge, this is the first study showing *PACSIN2* genotype association with hematological toxicity in ALL patients undergoing maintenance therapy.

Cure rates of approximately 80% can currently be achieved in paediatric acute lymphoblastic leukaemia (ALL)^1^,^2^. The overall success of treatment can, in part, be attributed to adequate maintenance therapy consisting of daily oral 6-mercaptopurine (6-MP) and a weekly low dose of methotrexate (MTX), necessary for retaining durable remission^3^. Drug-related toxicities during the maintenance phase of treatment remain one of the major concerns, not only because they can be life threatening, but also because they are a major cause of treatment interruption, which can result in an increased risk of relapse^4^.

6-MP is a prodrug that exerts its cytotoxic effects after several steps of metabolic activation leading to the formation of 6-thioguanine nucleotides (6-TGN). The main inactivating pathways are through xanthine oxidase (XO), which metabolizes 6-MP to thiouric acid, and through thiopurine S-methyltransferase (TPMT) metabolizing 6-MP to 6-methylmercaptopurine (6-MMP). The large variations in responses to 6-MP therapy that have been observed between patients are, to a great extent, attributed to differences in TPMT activity that lead to different accumulations of 6-TGNs in cells^5^. Patients exhibiting decreased TPMT activity have been shown to be at greater risk of experiencing toxic effects, whereas ultra-high enzyme activity can lead not only to superior 6-MP tolerability but also to an increased risk of relapse^6^. Genetic polymorphisms in *TPMT* can explain the majority, but not all, of the cases exhibiting decreased TPMT activity in individuals. Due to an incomplete genotype-to-phenotype correlation, which can be as low as 50% in heterozygotes in some populations^7^,^8^, it has now been well recognized that there are other genetic and non-genetic factors influencing TPMT activity. Two novel factors have recently been associated with TPMT, one of which is the non-genetic factor S-adenosylmethionine (SAM), the principal cellular methyl donor and TPMT co-substrate. Its influence on TPMT activity has been demonstrated in molecular and functional studies *in vitro* on several cancer cell lines^9^,^10^ and *in vivo* in a study involving over 1,000 healthy individuals^11^. The second factor is genetic variation in *PACSIN2*, which has been found to be associated with TPMT activity and with the incidence of 6-MP-related gastrointestinal toxicity^12^.

Since SAM is synthesized in the methionine cycle, which is closely related to one-carbon metabolism, its intracellular concentration depends on the availability of methionine as well as the folate pool. Based on this knowledge, we hypothesized that polymorphisms in genes coding for enzymes involved in the methionine and folate pathways, such as are presented in Fig. 1, might influence 6-MP toxicity in treated patients. We have previously shown that polymorphisms in methylenetetrahydrofolate reductase (MTHFR) augment the effect of TPMT heterozygosity on 6-MP related toxicities^13^. The aim of this study was to investigate the association of 6-MP related toxicities and other selected polymorphisms of the folate pathway, together with polymorphisms in *PACSIN2* and *ITPA*, which have previously been associated with treatment toxicity^12^,^14^ in children with ALL undergoing 6-MP treatment. In order to exclude as much as possible the influence of other drugs used in ALL treatment on 6-MP related toxic effects, we focused on the maintenance phase of the therapy, because it consisted exclusively of 6-MP and low dose MTX.

## Results

### Patients and genotyping

In total, 305 paediatric ALL patients with a mean age of 5.9 ± 4.3 years were included in the study. Their clinical characteristics are presented in Table 1.

We analysed 11 SNPs in 8 candidate genes, all of which showed distribution in accordance with the Hardy–Weinberg equilibrium. The list of analysed SNPs with information on variant allele frequencies and p-values of Hardy-Weinberg tests are presented in Table 2.

Hematotoxicity, stomatitis, and recurrent infections, except secondary tumours, were in correlation to each other as well as to 6-MP dose reduction (Chi-square test, p < 0.05). These observations are expected, since dose was usually reduced as a consequence of experienced toxicity. Furthermore, it was not uncommon for the same patient to experience more than one adverse drug effect, due to overall worse tolerability of treatment.

### Multilocus genotypes associated with 6-MP related hematotoxicity and 6-MP dose reduction

During the period of maintenance therapy, hematotoxicity of grade 3 or 4 was experienced by 15% (46) of patients while 6-MP dose reduction (at least a 10% reduction lasting 3 months or more) was recorded in 20% (63) of patients.

In order to assess the effects of the candidate polymorphisms and their interaction in predisposing the patient to 6-MP induced hematotoxicity and dose reduction, we used multivariate logistic regression models along with generalized multifactor dimensionality reductions (GMDR) and classification and regression tree (CART) analyses.

**Figure 2:**
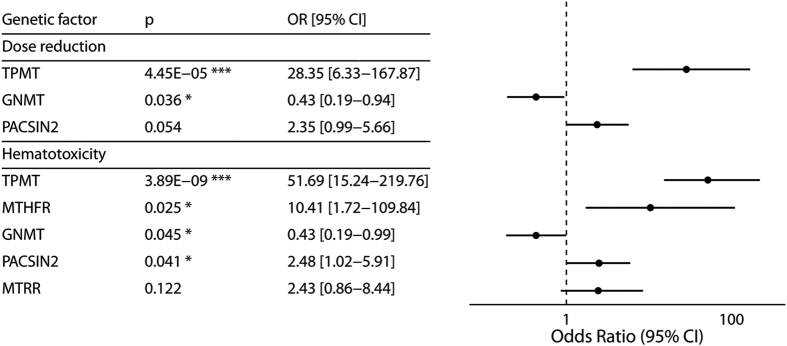
Effects of genotypes in multivariate logistic regression models predicting dose reduction and occurrence of 6-MP related hematotoxicity. Footnotes: Significance codes (p): ***0.001, **0.01, *0.05, “.” 0.1; Abbreviations: OR = odds ratio, CI = confidence interval; Both models were adjusted to treatment protocol, age at diagnosis and gender. Genetic factors: TPMT: *1/*3 vs. *1/*1; GNMT: 1298CC vs 1298CT/TT; PACSIN2: rs2413739 CC/CT vs rs2413739 TT; MTHFR: wild-type (677CC/1298AA) vs genotype combinations with at least one mutation (677CC/1298AC, 677CT/1298AA, 677CC/1298CC, 677TT/1298AA, 677CT/1298AC, 677TT/1298AC); MTRR: 66AA vs 66AG/GG.

As indicated in Fig. 2, the following genetic factors were included in the multivariate logistic model for hematotoxicity: TPMT and MTHFR, both of which have been already described^13^, as well as GNMT, PACSIN2 and MTRR genotypes. The analysis revealed a lower risk of experiencing hematotoxicity in patients with *GNMT* 1298CT or the TT genotype than in those with *GNMT* 1298CC (OR = 0.43; 95% CI = 0.19–0.99; p = 0.045) and a higher risk in patients with *PACSIN2* TT than in those with *PACSIN2* CC or the CT genotype (OR = 2.48; 95% CI = 1.02–5.91; p = 0.041). The effect of the MTRR genotype was not statistically significant and neither were the effects of other genotypes, except for TPMT, after Bonferroni correction.

**Figure 3:**
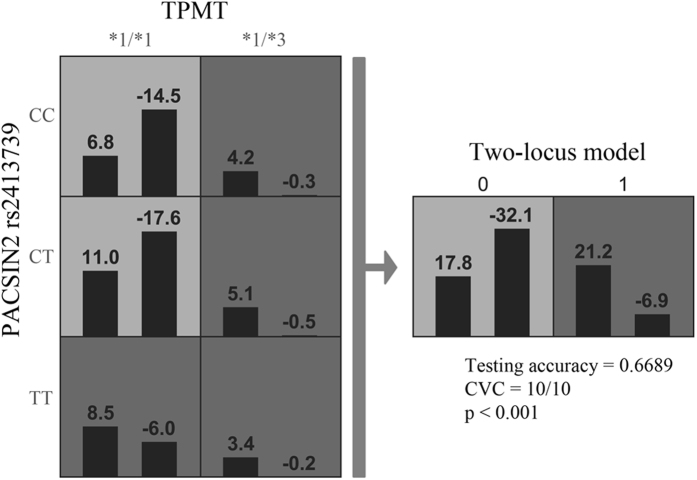
The result of the best overall GMDR model for hematotoxicity after adjustment for treatment protocol, age at diagnosis and gender. The plot shows in each cell a positive score (left bars) and a negative score (right bars), which was calculated in the GMDR model after treatment protocol, gender and age at diagnosis adjustment for each genotype combination. Based on determined score statistics patients in each cell were assigned to have either a high-risk or low-risk genotype (shaded dark grey and light grey, respectively). TPMT *1/*3 denotes a heterozygous genotype (*1/*3A or *1/*3C). In the two-locus model “0” presents low risk group (patients with wild-type TPMT and PACSIN2 rs2413739 CC or CT) and “1” presents high-risk group (patients with heterozygous TPMT genotype and wild-type TPMT patients with PACSIN2 rs2413739 TT genotype).

The best overall GMDR model for the prediction of hematotoxicity revealed a two-locus interaction between TPMT rs1142345/rs1800460 and PACSIN2 rs2413739. The model, adjusted to treatment protocol, age at diagnosis and gender, had a cross-validation consistency of 9/10 and a balanced testing accuracy of 0.6692 (Table 3). The result indicates that besides patients carrying variant TPMT allele, wild-type TPMT patients with the PACSIN2 rs2413739 TT genotype are at a higher risk of experiencing hematological toxicity than wild-type TPMT patients with the PACSIN2 rs2413739 CT or CC genotype (Fig. 3). The new genetic classifier, constructed based on the GMDR analysis, was subsequently entered in the multivariate logistic regression analysis to compute an odds ratio (OR), subsequently established as 6.18 (95% CI: 3.07–12.76, p = 4.85 * 10^−7^), when the model was adjusted to protocol treatment, age at diagnosis and gender. The effect remained significant after Bonferroni correction.

**Figure 4:**
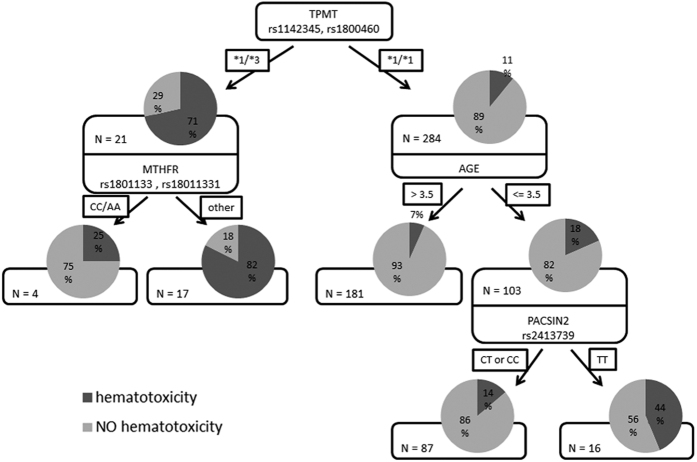
Results of CART analysis for risk of hematotoxicity. Each branch of the tree is divided by genotype and other co-variables based on the information gain criterion. The following information is presented in each node: distribution pie chart with target class probability where “hematotoxicity” class is represented in dark gray and “NO hematotoxicity” in light gray, and number of patients in each node.

Next, we conducted a CART analysis during which patients were subdivided successively by the most discriminating polymorphism or haplotype to form a so-called regression tree. The results of this are presented in Fig. 3. The initial split of the root node in CART analysis for hematotoxicity was the TPMT genotype, confirming that it is the strongest risk factor for 6-MP-induced hematotoxicity (Fig. 4). A deeper exploration of the classification tree structure demonstrated distinct interaction patterns in wild-type and variant TPMT individuals. From the classification tree (Fig. 4), it appears that the effect of the MTHFR genotype is more important in variant TPMT individuals, whereas the effect of the PACSIN2 rs2413739 TT genotype emerges in wild-type TPMT patients under the age of 4 years. The odds ratio for the new genetic classifier including PACSIN2 rs2413739 and age at diagnosis was estimated using the logistic regression analysis, confirming its strong association with hematotoxicity in a subset of patients with wild-type TPMT (OR = 7.80, 95% CI = 2.40–25.34, p = 0.0005). The model was adjusted to treatment protocol and gender and the effect remained significant after Bonferroni correction.

For dose reduction, the logistic regression model with the smallest value of AIC was that including TPMT, GNMT and PACSIN2 genotypes. Besides the influences of treatment protocol and TPMT genotype, described previously^13^, this model revealed an additional effect of the *GNMT* genotype on dose reduction, such that a lower frequency of dose reduction was observed in patients with *GNMT* 1298CT or TT than was the case in those with the *GNMT* 1298CC genotype (OR = 0.43; 95% CI = 0.19–0.94; p = 0.036). The effect did not remain significant after Bonferroni correction. *PACSIN2* genotype contributed to the overall fit of the model, but was not statistically significant (Fig. 2).

The GMDR analysis for dose reduction, adjusted for treatment protocol, age at diagnosis and gender, did not reveal any significant gene-gene interaction (Table 3). The best two-locus and three-locus models both had very low cross-validation consistencies; consequently, we did not consider them for further analysis.

**Figure 5:**
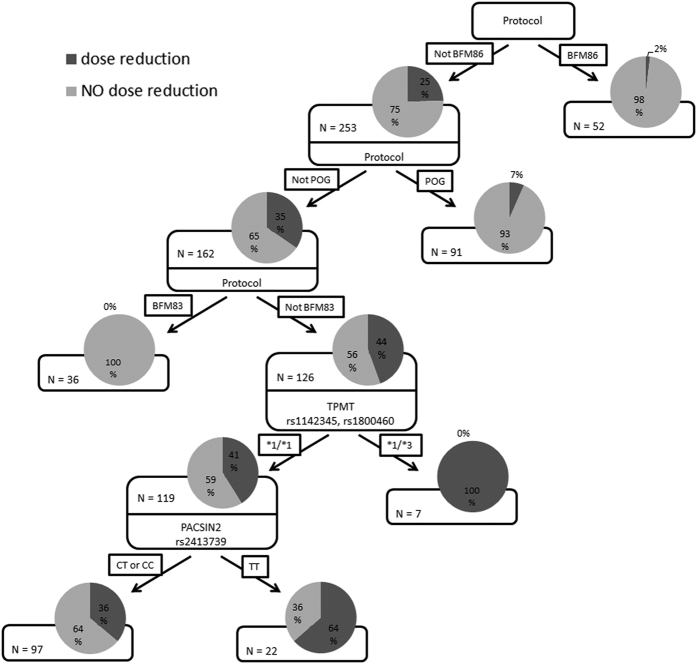
Results of CART analysis for dose reduction. Each branch of the tree is divided by genotype and other co-variables based on the information gain criterion. The following information is presented in each node: distribution pie chart with target class probability where “dose reduction” class is represented in dark gray and “NO dose reduction” in light gray, and number of patients in each node.

The CART analysis for dose reduction demonstrated the strongest effect of treatment protocol, where patients treated in accordance with older protocols (POG, BFM83 and BFM86) had their dose reduced less often than patients treated according to protocols BFM90, BFM95 or BFM2002. The reason for this is difference in the management of patients, where treating physicians working within the older protocols tended to discontinue 6-MP in the event of adverse events rather than lower the dose. The effect of genotype is, therefore, seen only in the more recent protocols, where the TPMT genotype again emerged as the strongest predictor of dose reduction. In wild-type TPMT patients, an additional influence of PACSIN2 rs2413739 emerged, with patients carrying the TT genotype being at a higher risk of dose reduction relative to patients with CC or CT genotypes (Fig. 5). The genetic classifier constructed on the basis of the CART analysis was used in a logistic regression model adjusted to treatment protocol, age at diagnosis, gender and TPMT genotype. The OR for the new classifier was 3.19 (95% CI: 1.26–8.80; p = 0.020), however, the effect did not remain statistically significant after Bonferroni correction.

### Multilocus genotypes associated with other 6-MP related toxicities

During the maintenance therapy period, infections of grade 3 or 4 and stomatitis of grade 2 or 3 were experienced by 22% (67) and 6% (19) of patients, respectively. 4% (12) of patients developed secondary tumours during or after the maintenance therapy.

In the logistic regression analysis, the addition of the MTRR genotype variable to the predictive model for stomatitis already including TPMT genotype improved the model, although the effect of this addition was not statistically significant (Figure S1). Further genotype information did not improve the predictive model for the occurrence of sepsis/recurrent infections, with age at diagnosis and TPMT genotype remaining the only statistically significant predictors for recurrent infections after Bonferroni correction (p = 0.00015) (Figure S1). None of the analysed variables demonstrated a statistically significant effect on the incidence of secondary tumours.

The best overall GMDR model for stomatitis suggested an interaction between MTRR rs1801394 and MTHFR rs1801133 polymorphisms (Table S1). The two-locus model had a CVC of 9/10 and a testing accuracy of 0.7568, but its significance after permutation testing was only marginal. Nevertheless, a new genetic classifier was constructed based on the analysis (Figure S2); it had a corrected OR of 4.09 (95% CI: 1.54–11.30, p = 0.005) in the multivariate logistic regression analysis adjusted to protocol treatment, age at diagnosis, gender and TPMT genotype and it remained statistically significant after Bonferroni correction.

As in the case of dose reduction prediction, the GMDR analysis for infections adjusted for treatment protocol, age at diagnosis and gender, established a one-locus model containing TPMT genotype information as the best overall model (Table S1). The two- and three- locus models both had very high CVC values and fairly good testing balanced accuracies, but did not prove to be statistically significant after a 1,000 permutation test was conducted.

No statistically significant predictive GMDR model was obtained in the analysis of secondary tumours.

No significant gene-gene interaction was detected with CART analysis in connection with the risk of stomatitis or infections (Figure S3A,B). The only interaction revealed was between age at diagnosis, TPMT genotype and protocol treatment. No gene-gene interaction in the form of a new genetic classifier was identified for CART analysis of secondary tumours.

## Discussion

In this retrospective study in paediatric ALL patients undergoing maintenance therapy, we analysed 11 polymorphisms in 8 candidate genes that could influence the metabolism of 6-MP, either by being directly involved in the metabolism itself or indirectly by altering TPMT activity. We investigated their associations and possible interactions in treatment-related toxicities through the application of a multi-analytical approach. Although each strategy used different algorithms to define these interactions, results from LR, CART, and GMDR consistently showed that the TPMT genotype was the most significant single risk factor for developing 6-MP related toxicity, and that its interaction with PACSIN2 rs2413739 was substantially associated with the risk of experiencing hematotoxicity.

It has long been recognized that TPMT genotype is the primary risk factor for **hematotoxicity** in patients undergoing 6-MP treatment and the same was indicated in the present study as the TPMT genotype showed a strong association in logistic regression, in the single best one-factor model in GMDR, and formed the first split in CART analysis. The TPMT genotype was also identified as the strongest single genetic predictor for sepsis/recurrent infections (by all methods), for stomatitis (by LR and CART methods) and was associated with the occurrence of dose reductions due to therapy toxicities. An interaction between the TPMT genotype and PACSIN2 rs2413739 was consistently identified by all statistical methods in the case of hematotoxicity; namely, that patients with wild-type TPMT who had the PACSIN2 rs2413739 TT genotype stood a higher risk of experiencing hematotoxicity than patients carrying the PACSIN2 rs2413739 CT or CC genotype. Similarly, the effect of the PACSIN2 genotype in wild-type TPMT patients was associated with dose reductions according to LR and CART analysis (although the statistical significance was lost after Bonferroni correction), but this association was not shown by GMDR.

PACSIN2 is a member of the ‘protein kinase C and casein kinase substrate in neurons’ family of proteins that are involved in vesicle formation via interactions with the large GTPase dynamin and N-WASP, which forms a critical part of the actin polymerisation machinery^15^. The only clinically relevant polymorphism in *PACSIN2* is rs2413739, which had previously only been associated with 6-MP-related gastrointestinal toxicity in ALL patients during consolidation therapy^12^. It was suggested by the researchers that the association of the *PACSIN2* genotype with 6-MP-related toxicity occurs through the modulation of TPMT activity, a hypothesis that was confirmed in *in vitro* cell models as well as in ALL patients, where individuals with the *PACSIN2* rs2413739 CC genotype had higher TPMT activity than patients with the TT genotype^12^. Another possible mechanism of PACSIN2’s influence on hematotoxicity could be through its interaction with Rac1, which has recently been reported^16^. It is known that one of the mechanisms of action of thiopurines proceeds by active metabolites – specifically, 6-thioguanine triphosphate (6-TGTP) – binding to Rac1, blocking its activation and, subsequently, inducing apoptosis^17^. The interaction of PACSIN2 with Rac1 could, therefore, cause an increase in the sensitivity of cells to 6-MP and, in turn, increase the patient’s risk of experiencing hematotoxicity.

In CART analysis age under 4 years emerged as the risk factor for hematotoxicity forming a split before PACSIN2 genotype in wild-type TPMT patients. However, since in LR model the effect of age was not statistically significant and we found no reference describing the same effect in the published literature, it is likely that this observation has no clinical relevance.

Of the genes related to the folate and methionine cycles that were hypothesised as having an effect on 6-MP-related toxicity by modulating SAM levels and, consequently, TPMT activity, only MTHFR was associated with hematotoxicity by two statistical methods (LR and CART), although the effect did not remain statistically significant after Bonferroni correction. This observation is in agreement with previously published results^13^,^18^. MTHFR genotype has long been established as an important risk factor for MTX-related toxicities in ALL^19^, however its observed effect on treatment-related toxicity in our study is more likely to be attributed to the possible TPMT modulating effect, as dose of MTX during the maintenance therapy is very low and thus less likely to contribute to toxic effects. The absence of any gene-gene interaction detected by the GMDR model might be attributed to differences in the coding of variables. In the GMDR analysis, separate SNPs were analyzed, whereas in the LR model only one MTHFR variable, which included information on both analyzed SNPs (wild-type MTHFR compared to MTHFR with at least one variant allele), was used. Although the grouping of functional SNPs in a single gene to one variable makes biological sense, the presence of at least one variant allele can result in decreased enzyme activity, such that the haplotype analysis can, in some cases, disturb the selection of the best genetic classifier, especially in GMDR analysis^20^. In our study we observed just such a case in the analysis of the risk of stomatitis. In the best-fit LR model, two genetic factors, namely TPMT and MTRR, were included; however, only the TPMT genotype was statistically significant. The inclusion of the MTRR genotype improved the overall model and suggested it had an influence, but did not prove its independent effect on the occurrence of stomatitis. On the other hand, an interesting gene-gene interaction was detected in the GMDR analysis, which is able to detect interactions even in the absence of a main effect^21^. A new genetic classifier, composed of MTRR rs1801394 and MTHFR rs1801133, was the best predictor of stomatitis and was proved to be statistically significant in the subsequent LR analysis, adjusted to treatment protocol, age at diagnosis, gender and TPMT genotype. None of the other polymorphisms studied was associated with hematotoxicity and dose reduction after Bonferroni correction.

A major strength of our study is that some gene-gene interactions, including, and in particular that between TPMT and PACSIN2, were consistently identified when analyzing data by different statistical approaches. The analysis was first done by logistic regression (LR), which is considered to be the workhorse of modern epidemiology^22^ and has the advantage of controlling for confounding variables simultaneously. However, when high-order interactions involving multidimensional factors are considered, the increased dimensionality may result in large standard errors and an increased risk of type I errors^23^. To complement logistic regression, we then applied the GMDR method, which is a nonparametric and genetic-model-free data mining strategy designed to detect interactions in the absence of detectable main effects^21^. The power to detect potential gene-gene interactions is increased by converting multiple variables to a single attribute while the chance of making type I errors is decreased by cross validation and permutation testing^21^. As an additional nonparametric test, we also used CART analysis, an explorative decision tree–based data mining approach that requires no assumption of a genetic model. Here, the genetic contribution to drug response is analysed taking into account that a polymorphism may only have an impact on drug response on condition that another polymorphism is present. Both GMDR and CART analyses result in genetic classifiers that are associated with the studied outcome. Since they rely on different ways of creating new genetic classifiers, these may not always contain the same polymorphisms. In our study, three statistical methods (LR, MDR, and CART) validate each other with regard to a combined TPMT and PACSIN2 association with the risk of hematotoxicity, thus emphasizing the reproducibility of our findings. On the other hand, the methods complement each other in the case of risk of stomatitis, where the GMDR analysis revealed an independent interaction between MTRR and MTHFR genotypes in the absence of the main effect and was successfully validated by LR method.

There are some limitations to our findings. Firstly, our study evaluated a limited number of patients and, due to the small number of cases, for example in the analysis of stomatitis and secondary tumours (only 19 and 12 cases in 305 patients, respectively), some risk factors might not have been picked up by the statistical analysis. In addition, patients included in our study were treated according to different treatment protocols over a relatively large period of time. Although the regimens of the maintenance phase of therapy were very similar in all the protocols and all our statistical models were adjusted to this covariable, the genetic effects detected in our study should be evaluated in an independent cohort of patients treated in accordance with the most recent treatment protocols so as to prove their clinical utility. Furthermore, we only investigated a limited number of polymorphisms in our study. Other genes, such as those related to the folate pathway, including TYMS and FOLH1^24^, could also contribute to the development of 6-MP-related toxicities, possibly by interacting with other genes regulating intracellular SAM levels.

In conclusion, although *PACSIN2* has previously been identified as influencing gastrointestinal toxicity during consolidation therapy, this is, to our knowledge, the first study showing its association with hematological toxicity in ALL patients undergoing maintenance therapy, a finding consistently detected by different statistical methods. We believe these results warrant further investigation of this and other genetic classifiers (including MTHFR and MTRR genotypes) in larger prospective studies to prove or reject their utility in improving the outcome of thiopurine therapy.

## Materials and Methods

### Study subjects

ALL patients diagnosed and treated at University Children’s Hospital, University Medical Centre, Ljubljana, Slovenia from 1970 to 2006 were identified through the National Cancer Registry. Of 408 registered patients with childhood ALL, adequate documentation and intact genetic material was obtained from 308 patients. Due to the unsuccessful genotyping of PACSIN2 rs2413739 in 3 patients, these were removed from subsequent analysis, such that the final study group consisted of 305 patients. The following therapy protocols were applied: from 1970 to 1983 the USA Paediatric Oncology Group (POG) protocols were used; from 1983 the therapy was changed to the German Berlin-Frankfurt-Muenster (BFM) protocols (BFM-83, −86, −90, −95 and IC2002). Maintenance therapy was included in all protocols and lasted from 1 to 3 years. The maintenance phase consisted of daily oral 6-MP (50 mg/m^2^) and weekly oral low-dose methotrexate (MTX) (20 mg/m^2^).

Patients’ characteristics, such as gender, age at diagnosis, therapy protocol and risk group allocation, were obtained from their medical records. Ethical approval for this study was obtained from the National Medical Ethics Committee of Slovenia (59/07/10) which waived the need for written informed consent, since the data was collected retrospectively and was analyzed anonymously. Methods were carried out in accordance with the approved guidelines.

### Toxicities and risk group definition

We have followed some of the most commonly observed toxic effects during the maintenance therapy, leading to discontinuation of the therapy for longer than one week, a reduction of over 10% of 6-MP dose for a duration greater than 3 months, or hospitalization of the patient and graded them using National Cancer Institute Common Toxicity Criteria (version 2.0), which was valid at the time of medical data collection and classification. Since the criteria for grading leukopenia, stomatitis and secondary tumours in the latest version of toxicity criteria (CTCAE 4.03) have not changed, results would apply also if new toxicity criteria were used. However, we acknowledge the possibly different results when analysing occurrence of infections, since grading differs between the two versions. For the analyses conducted, toxicity grades were used to dichotomize toxicities as “present” versus “absent”. Hematotoxicity corresponding to grade 3 and 4 leukopenia, infections of grade 3 to 4, stomatitis of grade 2 to 3 and secondary tumours of grade 4 were classified as present, while lower grades were considered absent. In addition, associations of polymorphisms and occurrences of dose reductions were investigated. Dose reduction was classified as present when the 6-MP dose was reduced by more than 10% and was of a duration greater than 3 months.

Patients were stratified into risk groups according to criteria described previously^25^,^26^,^27^,^28^. The standard risk group included the low-risk group of POG, the SR-L and SR-H groups of BFM-83 and SR groups of protocols BFM-86, −90 and −95. Intermediate or high-risk groups included the high-risk group of POG, the R-group of BFM-86, MR groups of BFM-83, −90, −95 and HR groups of the BFM-83, −90, −95 protocols.

### DNA extraction and genotyping

DNA extraction was performed as previously described^13^,^18^. All the analysed polymorphisms were determined by means of TaqMan chemistry using either the ABI Prism 7000 Sequence Detection system or the Roche LightCycler 480 system, in accordance with the manufacturers’ instructions. The amount of DNA used in each individual assay was 10 ng. The TaqMan SNP Genotyping Assay part numbers are available in ref. 29.

### Statistical analysis

The distributions of genotypes and possible deviations from the Hardy-Weinberg equilibrium were assessed using Fisher’s exact test. Odds ratios, 95% confidence intervals (95% CI) and p values, showing the association of the occurrence of adverse effects with studied genotypes, were calculated using logistic regression analysis. All models were adjusted to treatment protocol, age at diagnosis and gender. In addition, for each of the toxicities, models were fit for all possible subsets of covariates, including all of the determined genotypes. The models were then compared using the Akaike Information Criterion (AIC) in order to determine the ‘best’ prognostic model based on the data^30^. The model with the smallest AIC value (representing the best fit) was selected for interpretation of results.

In order to explore the independent and synergistic effects of the studied SNPs on the toxicities, we performed a generalized multifactor dimensionality reduction (GMDR) analysis^31^ and Classification and regression tree (CART) analysis, both of which result in new genetic classifiers. The GMDR method is an extension of original multifactor dimensionality reduction (MDR)^32^, which, in contrast to MDR, allows adjustment for discrete as well as continuous variables. All the models in our analysis were, therefore, adjusted for protocol treatment, age at diagnosis and gender. The best model was chosen based on cross-validation consistency (CVC ≥8/10) and the highest testing balanced accuracy, which is the function of the percentage of true positives (TP), true negatives (TN), false positives (FP), and false negatives (FN) as defined as (TP + TN)/(TP + TN + FP + FN). Permutation testing was used to control for using multiple hypothesis testing. 1000 permutation replications were performed so as to determine the statistical significance of the best model^33^.

For the execution of the CART analysis, polymorphisms as well as haplotypes were used. The maximum tree depth was set to five levels. Attribute selection was based on the information gain (or entropy reduction) criterion. Pruning was set to a maximum depth of 5 levels and splitting was stopped when there were fewer than 10 instances in a node. Leaves with the same majority class were set to recursively merge and post-pruning with an m-estimate (m = 2) was set.

Statistical analyses were performed using R (logistic regression), Orange 2.7 (CART)^34^ and GMDR v09 with accompanying Perl script for permutation testing (for GMDR analysis)^35^. In order to correct for multiple testing, we have applied the most conservative approach by Bonferroni correction. Since we have tested 9 logistic regression models (5 initial for each toxicity and 4 additional tests of new genetic classifiers), a p-value threshold of 0.0056 was considered statistically significant after Bonferroni correction in all the corresponding tests.

## Additional Information

**How to cite this article**: Smid, A. *et al*. *PACSIN2* polymorphism is associated with thiopurine-induced hematological toxicity in children with acute lymphoblastic leukaemia undergoing maintenance therapy. *Sci. Rep.* **6**, 30244; doi: 10.1038/srep30244 (2016).

## Supplementary Material

Supplementary Information

## Figures and Tables

**Figure 1:**
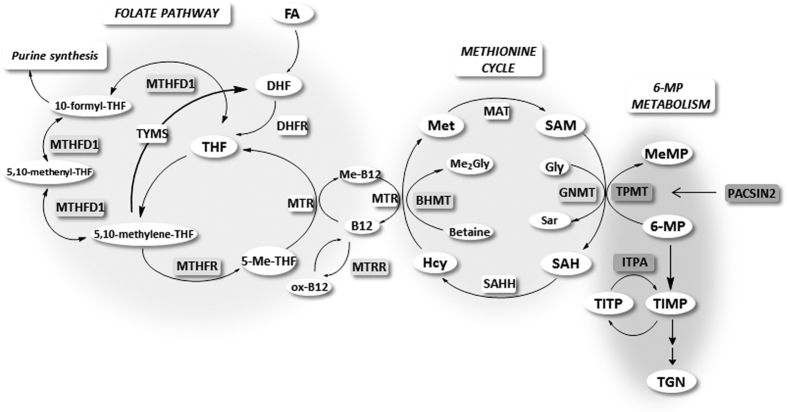
Scheme of the folate pathway, the methionine cycle and 6-MP metabolism. 6-MP, 6-mercaptopurine; BHMT, betaine-homocysteine S-methyltransferase; DHF, dihydrofolate; DHFR, dihydrofolate reductase; FA, folic acid; Gly, glycine; GNMT, glycine N-methyltransferase; Hcy, homocysteine; ITPA, inosine triphosphate pyrophosphatase; MAT, methionine adenosyltransferase; ox-B12, oxidised form of vitamin B12; Me2Gly, dimethyl-glycine; MeMP, methylmercaptopurine; Met, L-methionine; 5-Me-THF, 5-methyltetrahydrofolate; 5,10-methylene-THF, 5,10-methylenetetrahydrofolate; 5,10-methenyl-THF, 5,10-methenyltetrahydrofolate; MTHFD1, methylenetetrahydrofolate dehydrogenase (NADP + Dependent) 1; MTHFR, 5,10-methylenetetrahydrofolate reductase; MTR, 5-methyltetrahydrofolate-homocysteine methyltransferase; SAH, S-adenosyl-L-homocysteine; SAHH, S-adenosyl-L-homocysteine hydrolase; MTRR, 5-methyltetrahydrofolate-homocysteine methyltransferase reductase; SAM, S-adenosyl-L-methionine; Sar, sarcosine; THF, tetrahydrofolate; TGN, thioguanosine; TIMP, thioinosine monophosphate; TITP, thioinosine triphosphate, TPMT, thiopurine S-methyltransferase; TYMS, thymidylate synthetase.

**Table 1:** Clinical characteristics of patients with childhood ALL (N = 305).

Characteristic	No	%
Gender		
Female	142	46.6
Male	163	53.4
Mean age at diagnosis ± standard deviation		5.9 ± 4.3 years
ALL therapy protocol		
POG	91	29.8
BFM 83	36	11.8
BFM 86	52	17.0
BFM 90	58	19.0
BFM 95	55	18.0
IC-BFM 2002	13	4.3

Abbreviations: POG, Paediatric Oncology Group; BFM, Berlin-Frankfurt-Muenster.

**Table 2:** List of analysed SNPs with variant allele frequencies and testing for Hardy-Weinberg distribution.

Gene	SNP ID	Nucleotide change	Variant allele frequency	Hardy-Weinberg test (p-value)
TPMT	rs1800460	460G > A*	0.03	0.595
TPMT	rs1142345	719A > G*	0.03	0.534
MTHFR	rs1801133	677C > T	0.35	0.479
MTHFR	rs1801131	1298C > A	0.33	0.626
MTRR	rs1801394	66A > G	0.58	0.214
MTHFD1	rs2236225	1958G > A	0.45	0.626
BHMT	rs3733890	742G > A	0.33	0.106
GNMT	rs10948059	1298C > T	0.49	0.098
PACSIN2	rs2413739	C > T	0.41	0.322
ITPA	rs1127354	94C > A	0.06	0.220
ITPA	rs7270101	IVS2 + 21A > C	0.13	0.942

^*^All 18 patients carrying rs1800460 variant (A) were also carriers of rs1142345 variant (G) and were therefore defined as carriers of *3A haplotype. The remaining 3 patients carrying only rs1142345 variant (G) were defined as carriers of *3C and none as carrier of *3B.

**Table 3:** Results of the generalized multifactor dimensionality reduction (GMDR) analysis for hematotoxicity and dose reduction.

Toxicity	Best candidate model (for up to 3 factor combinations)†	Testing balanced accuracy	Cross validation consistency	Permutation testing P-value ‡
Hematotoxicity	TPMT	0.6363	10/10	<0.001***
	**TPMT, PACSIN2**	**0.6692**	9/10	0.006**
	MTRR,MTHFD,PACSIN2	0.6003	5/10	0.046
**Dose reduction**	**TPMT**	**0.6018**	10/10	0.001**
	GNMT, BHMT	0.6149	5/10	0.024*
	MTHFD, BHMT, PACSIN2	0.5787	4/10	0.107

^†^All models were adjusted for treatment protocol, age at diagnosis and gender. Permutation testing was performed with 1,000 permutation repetitions.

^‡^Significance codes (p): ***0.001, **0.01, *0.05.
