# Supplementary material for: PACSIN2 polymorphism is associated with thiopurine-induced hematological toxicity in children with acute lymphoblastic leukaemia undergoing maintenance therapy
Source: Sci Rep. 2016 Jul 25;6:30244. doi: 10.1038/srep30244 (PMC4958958; doi:10.1038/srep30244)
Supplement: Supplementary Information [file srep30244-s1.pdf]

PACSIN2 polymorphism is associated with thiopurine-induced haematological toxicity in children with acute lymphoblastic leukaemia undergoing maintenance therapy

Alenka Smid[1], Natasa Karas-Kuzelicki[1], Janez Jazbec[2], Irena Mlinaric-Rascan[1,*]

[1]Faculty of Pharmacy, University of Ljubljana, Ljubljana, Slovenia;
[2]University Children's Hospital, University Medical Centre Ljubljana, Ljubljana, Slovenia

*Corresponding author: Irena Mlinaric-Rascan, PhD, Faculty of Pharmacy, University of Ljubljana, Askerceva 7, SI-1000 Ljubljana, Slovenia. Tel.: +386(0)14769645, Fax: +386(0)14258031. E-mail: irena.mlinaric-rascan@ffa.uni-lj.si

SUPPLEMENTARY RESULTS

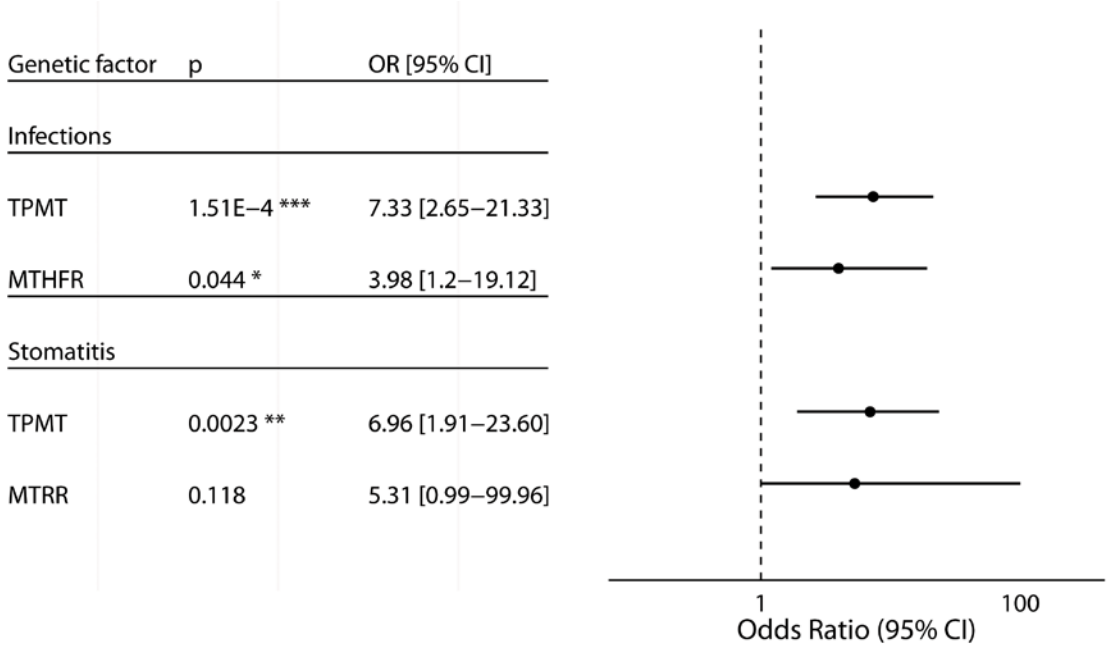

Figure S1. Effects of genotypes in multivariate logistic regression models predicting recurrent infections and occurrence of 6-MP related stomatitis.
Significance codes (p): *** 0.001, ** 0.01, * 0.05, "." 0.1;
Abbreviations: OR = odds ratio, CI = confidence interval;
Both models were adjusted to treatment protocol, age at diagnosis and gender.
Genetic factors: TPMT: *1/*3 vs. *1/*1; MTHFR: wild-type (677CC/1298AA) vs genotype combinations with at least one mutation (677CC/1298AC, 677CT/1298AA, 677CC/1298CC, 677TT/1298AA, 677CT/1298AC, 677TT/1298AC); MTRR: 66AA vs 66AG/GG

Table S1. Results of the generalized multifactor dimensionality reduction (GMDR) analysis for infections and stomatitis

Toxicity	*Best candidate model (for up to 3 factor combinations)†*	*Testing balanced accuracy*	*Cross validation consistency*	*Permutation testing P-value ‡*
**Stomatitis**	MTRR	0.5861	6/10	0.354
	**MTRR, MTHFR677**	**0.7568**	**9/10**	**0.081**
	MTRR, MTHFR 677, PACSIN2	0.7229	6/10	0.142
**Infections**	**TPMT**	**0.5764**	**10/10**	**<0.001*****
	MTRR, PACSIN2	0.574	9/10	0.071
	MTRR, BHMT, PACSIN2	0.5718	10/10	0.095

†All models were adjusted for treatment protocol, age at diagnosis and gender. Permutation testing was performed with 1000 permutation repetitions
‡ Significance codes (p): *** 0.001, ** 0.01, * 0.05

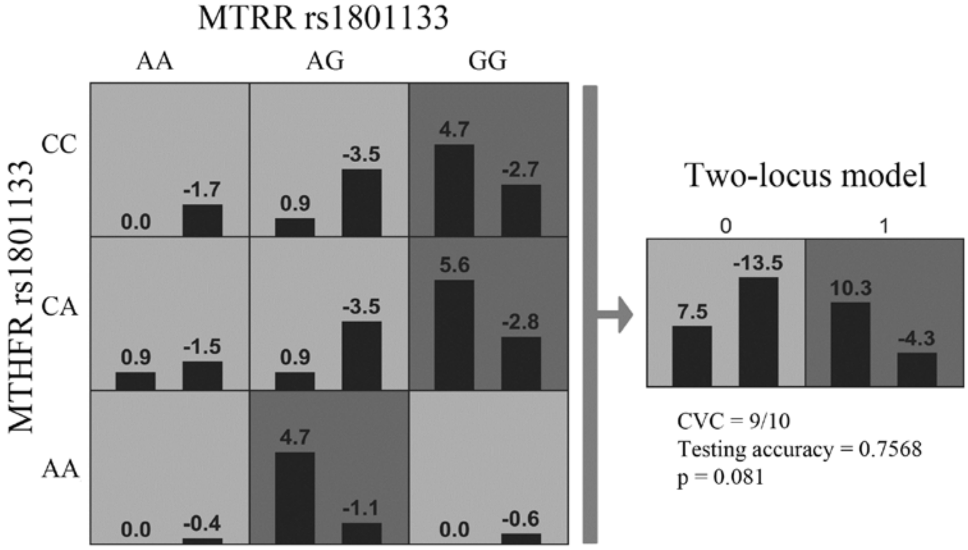

Figure S2. The result of the overall best GMDR model for stomatitis after adjustment for treatment protocol, age at diagnosis and gender.

The plot shows the in each cell a positive score (left bars) and a negative score (right bars), which was calculated in GMDR model after treatment protocol, gender and age at diagnosis adjustment for each genotype combination. Based on determined score statistics patients in each cell were assigned to have either a high-risk or low-risk genotype (shaded dark grey and light grey, respectively). The new genetic classifier (on the right) demonstrated an independent, significant effect in the logistic regression model adjusted for protocol, gender and age at diagnosis and TPMT genotype (OR = 4.09, 95% CI: 1.54 – 11.30, p = 0.005). The effect remained significant after Bonferroni correction.

A. **Risk for stomatitis**

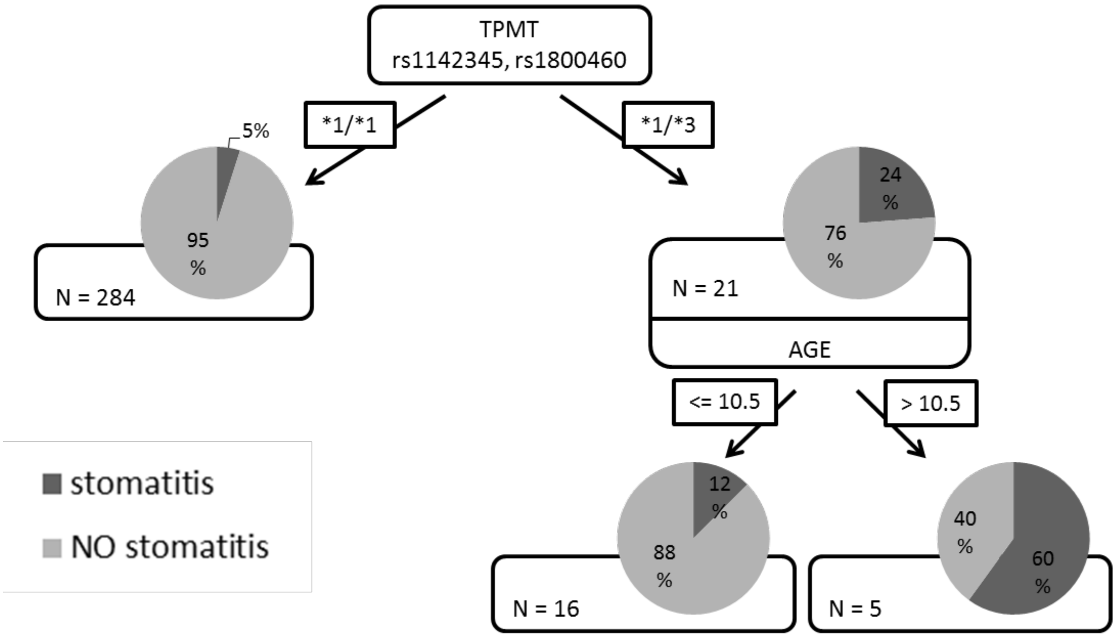

B. **Risk for infections**

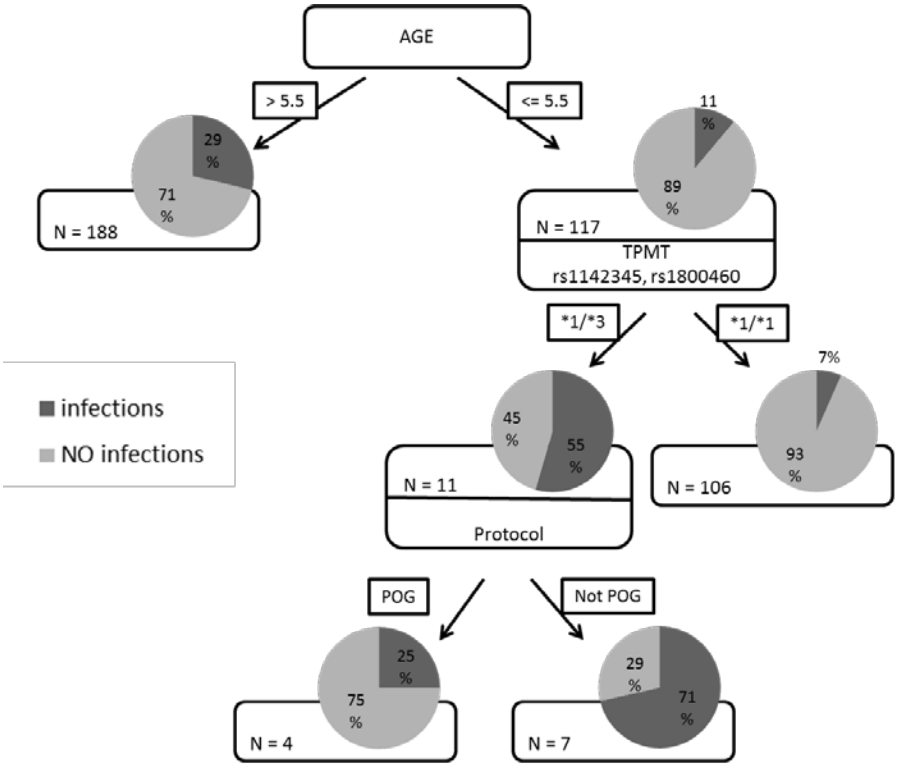

Figure S3. Results of CART analysis for stomatitis (A) and infections (B). Each branch of the tree is divided by genotype and other co-variables based on the information gain criterion. The following information is presented in each node: distribution pie chart with target class probability where "stomatitis/infections" class is represented in dark grey and "NO stomatitis/NO infections" in light grey, and number of patients in each node.
